# Cardiac risk and myocardial fibrosis assessment with cardiac magnetic resonance in patients with myotonic dystrophy

**DOI:** 10.3389/fneur.2024.1493570

**Published:** 2024-11-21

**Authors:** Elena Abati, Claudia Alberti, Valentina Tambè, Anastasia Esseridou, Giacomo Pietro Comi, Stefania Corti, Giovanni Meola, Francesco Secchi

**Affiliations:** ^1^Department of Pathophysiology and Transplantation, Dino Ferrari Center, Università degli Studi di Milano, Milan, Italy; ^2^Neurology Unit, Foundation IRCCS Ca’ Granda Ospedale Maggiore Policlinico, Milan, Italy; ^3^Radiology Unit, Casa di Cura Igea, Milan, Italy; ^4^Neuromuscular and Rare Diseases Unit, Foundation IRCCS Ca’ Granda Ospedale Maggiore Policlinico, Milan, Italy; ^5^Department of Neurorehabilitation Sciences, Casa Di Cura Igea, Milan, Italy; ^6^Department of Biomedical Sciences for Health, Università degli Studi di Milano, Milan, Italy; ^7^Unit of Cardiovascular Imaging, IRCCS MultiMedica, Milan, Italy

**Keywords:** myotonic dystrophy, extracellular volume, sudden cardiac death, conduction disturbances, imaging biomarkers

## Abstract

**Introduction:**

Non-invasive evaluation of myocardial tissue is a major goal of cardiac imaging. This is the case of myocardial fibrosis which is crucial in many myocardial diseases. Cardiac extracellular volume (ECV) was shown to indicate myocardial fibrosis and early cardiac involvement. With this study, our objective is to evaluate ECV measured with cardiac magnetic resonance (CMR) in patients with myotonic dystrophy type 1 (DM1) and 2 (DM2) as potential imaging biomarkers of subclinical cardiac pathology, and its relationship with demographic and clinical parameters, ECG-derived measures of cardiac conduction, and neuromuscular performance status.

**Materials and methods:**

We retrospectively analyzed 18 DM1 patients and 4 DM2 patients without apparent cardiac disease who had CMR at our center. Differences between independent distributions were evaluated using Mann–Whitney U test, while correlations were evaluated using Spearman’s *ρ*.

**Results:**

Global ECV in DM1 patients (median 28.36; IQR 24.81–29.77) was significantly higher (*p* = 0.0141) than in DM2 patients (median 22.93; IQR 21.25–24.35), and than that reported in literature in healthy subjects (*p* = 0.0374; median 25.60; IQR 19.90–31.90). Septal ECV was significantly higher (*p* = 0.0074) in DM1 (median 27.37; IQR 25.97–29.74) than in DM2 patients (median 22.46; 21.57–23.19). Global ECV showed a strong, positive correlation with septal ECV (*ρ* = 0.9282, *p* < 0.0001). We observed that DM1 women showed significantly higher global (*p* = 0.0012) and septal (*p* < 0.0001) ECV values compared to men.

**Discussion:**

We found a significant increase in global and septal cardiac ECV in patients with DM1. These values might thus suggest that DM1 patients present an increased cardiovascular risk, mainly due to cardiac fibrosis, even in absence of overt cardiac pathology at other common cardiovascular exams. DM1 patients may also be at increased risk of early septal fibrosis, with important implications on the risk for fatal arrhythmias. In addition, our results suggest the presence of gender-related differences, with DM1 women being more prone to myocardial fibrosis. Physicians dealing with DM1 may consider CMR as a screening tool for the early identification of patients with increased cardiovascular risk.

## Introduction

Myotonic dystrophy (DM), encompassing types 1 (DM1) (OMIM #160900) and 2 (DM2) (OMIM #602668), represents a group of autosomal dominant, multisystemic disorders characterized by muscle weakness and myotonia (1). DM1, caused by a *CTG* trinucleotide repeat expansion in the *DMPK* gene, affects approximately 1 in 8,000 individuals globally (2). DM2, although less common, shares similar clinical features but stems from a *CCTG* repeat expansion in the *CNBP* gene (3). Cardiac complications, particularly dysrhythmias, are prevalent in DM, particularly in DM1, where they are implicated in up to 80% of patients and stand as a leading cause of mortality, following respiratory complications (4). While the majority of neuromuscular disorders may cause cardiac dilation and cardiomyopathies, patients with myotonic dystrophies are at high risk also of conduction disturbances, which may have sudden and fatal presentations (5). The precise mechanisms by which DM1 promotes cardiac conduction system dysfunction are not well understood but may involve abnormal splicing of the *SCN5A* gene and upregulation of NKX2.5 (6, 7). From this perspective, DM as a neuromuscular disease is unique, presenting with a cardiac dysrhythmia risk requiring special management (4). Current clinical guidelines advocate for regular cardiac monitoring of DM patients, suggesting surface 12-lead ECG and cardiac imaging in every DM patient at baseline and annually if asymptomatic. Recommended imaging modalities, according to the American Heart Association, are echocardiography with strain rate imaging and CMR, and they are both acceptable (4). Up to now, echocardiography is usually the preferred modality given its wide availability and lower cost compared to CMR.

Despite these measures, traditional diagnostic tools may fail to detect early myocardial changes, particularly diffuse myocardial fibrosis, which can precede clinically evident cardiac manifestations (8). Diffuse fibrosis is characterized by increased collagen deposition and is a significant predictor of adverse cardiac outcomes as it can cause abnormal myocardial stiffness and contraction, eventually leading to heart failure (9). Therefore, assessing and quantifying diffuse fibrosis serves as a valuable biomarker for various cardiac diseases, offering the potential for early diagnosis and prognostic insight. This is true also for DM patients. Previous autopsy studies conducted in DM1 patients have uncovered fibrosis and fatty infiltration within the myocardium, suggesting that myocardial fibrosis is an underrecognised contributor to cardiac morbidity in this population (10–13). However, ECG and echocardiography primarily identify manifest arrhythmias and cardiomyopathies and may thus miss subclinical myocardial involvement (4).

Previously, detecting collagen buildup was only feasible through invasive myocardial biopsy. More recently, cardiac magnetic resonance (CMR) has proven useful in extending the spectrum of detectable, more subtle phenotypes (14). In particular, the analysis of late gadolinium enhancement (LGE) and the calculation of extracellular volume (ECV) with T1-mapping allow the detection of focal and diffuse fibrosis (9, 15). LGE seems to be more effective in the detection of focal fibrosis, while diffuse fibrosis may go undetected (15). Conversely, ECV measurement may identify diffuse fibrosis, which is present at an earlier disease stage. ECV is calculated from native T1 values, which are a composite signal deriving from myocytes and ECV; thus, the two primary biological factors which contribute to an elevation in native T1 are edema (an increase in tissue water content, as seen in acute ischaemia or inflammation) and the expansion of the interstitial space due to fibrosis (15). The most widely used CMR sequences to measure ECV are Modified Look-Locker Inversion Recovery (MOLLI) pulse sequences, which uses three consecutive inversion recovery pulses to acquire data from 17 breath-hold heart beats and to generate single-slice T1 maps of the myocardium (16, 17).

Previous studies have already documented CMR alterations pointing out at the presence of myocardial fibrosis in DM1 patients, such as LGE (18–23) and increase in ECV (24–27) (Table 1). Only few studies assessed ECV values in DM2 patients, showing lower values compared to DM1 (27) and no significant differences compared to controls (28, 29). Some studies only assessed patients without known cardiac disease, while other studies evaluated patients with a cardiac disease or conduction disturbance history (Table 1).

**Table 1:** Summary of previous literature studies assessing extracellular volume in patients affected by Myotonic Dystrophy type 1 and/or 2.

References	Number of patients	Age range	ECV values in DM patients	*p*-value compared to controls	Method	Instrument	Cardiovascular status
(26)	34 (DM1)	45 ± 12	33 ± 2	N/A	MOLLI	1.5T Signa CVi, GE Healthcare, Milwaukee, USA	38% patients had a history of AV block, 88% an intraventricular conduction disturbance, 12% an atrial fibrillation or flutter
(18)	57 (DM1)	43 ± 13	26 ± 3	0.57	MOLLI	1.5T Aera, Siemens Medical Solutions, Erlangen, Germany; Achieva, Philips, Best, The Netherlands	Known cardiac disease in 30% of patients
(25)	52 (DM1)	41 ± 14	25 ± 3	N/A	MOLLI	1.5T MAGNETOM Avanto, Siemens Healthineers, Erlangen, Germany	Conduction abnormalities in 60% of patients
(27)	13 (DM1)	45 ± 6	35.4 ± 10.8	DM1 vs. DM2: 0.0474	MOLLI	Ingenia 1.5 Tesla; Philips Healthcare	Only patients without known cardiac disease
	22 (DM2)		28.4 ± 6.4				
	Total DM cohort (35)		31.2 ± 8.9	0.0017			
(24)	9 (DM1)	36 (IQR 29–44)	32.3	0.008	MOLLI	1.5T MAGNETOM Avanto, Siemens Healthineers, Erlangen, Germany	Only patients without known cardiac disease
(29)	20 (DM2)	54 ± 10.6	26.1 ± 3% (basal)	0.28	MOLLI	1.5T scanner (MAGNETOM AvantoFit^®^, Siemens Healthineers, Erlangen, Germany)	Only patients without known cardiac disease
(28)	31 (DM2)	58 ± 9	26 ± 3 (basal)	N/A	MOLLI	1.5T scanner (MAGNETOM AvantoFit^®^, Siemens Healthineers, Erlangen, Germany)	72.7% asymptomatic, 27.3% reported palpitations, 4.5% chest pain, 22.7% fatigue

DM, myotonic dystrophy; ECV, extracellular volume; MOLLI, modified Look-Locker inversion recovery; N/A, not available.

Our study aims to further explore the potential of CMR, specifically through the evaluation of ECV, as a biomarker for subclinical cardiac pathology in patients with DM1 and DM2.

## Materials and methods

### Study design and population

**Figure 1:**
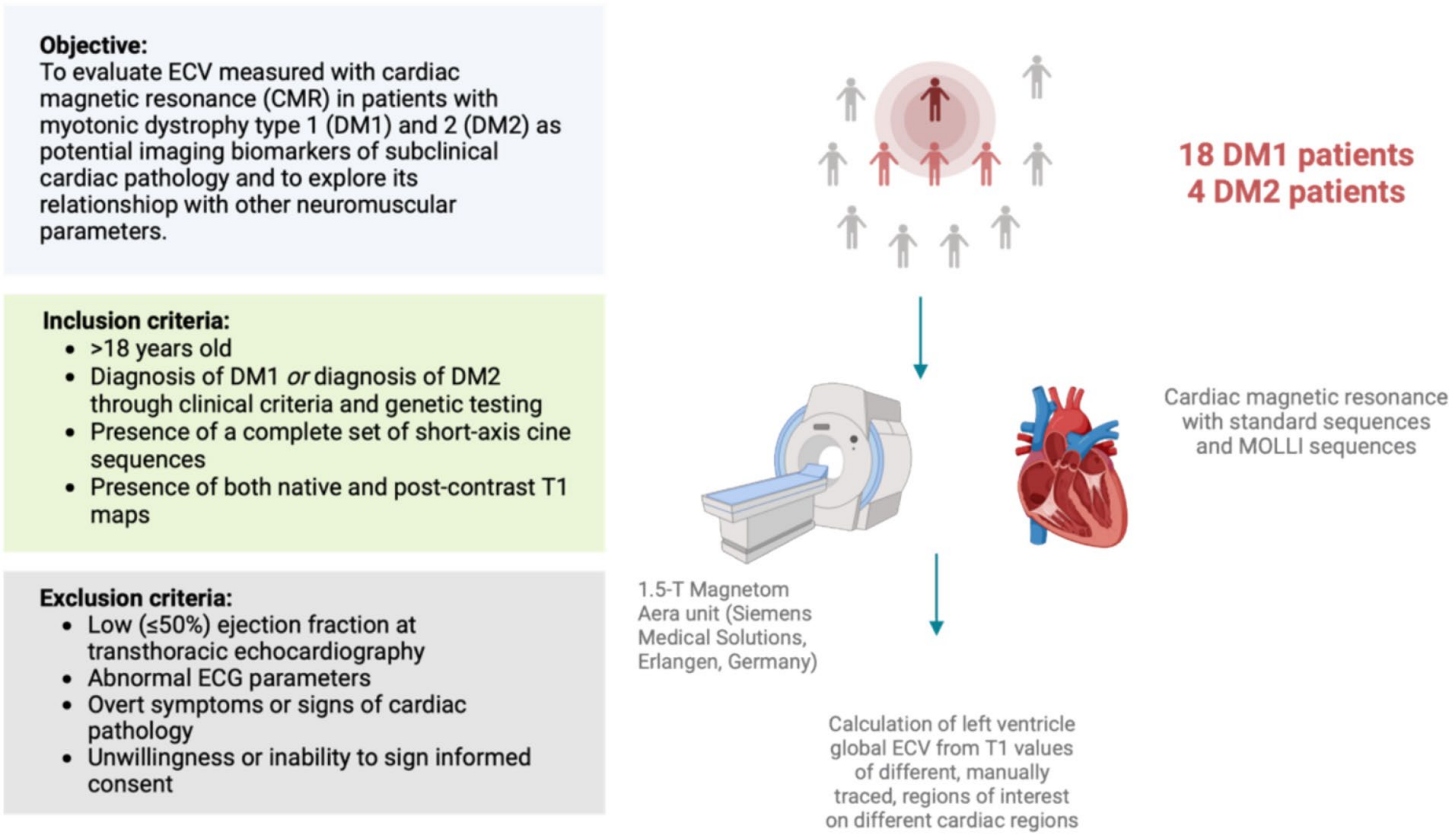
Schematic representation of study design.

We retrospectively analyzed images of DM1 and DM2 patients, without overt cardiovascular symptoms or disease, who had undergone a CMR examination as routine screening test for early cardiac involvement from March to October 2023 (Figure 1). Additional inclusion criteria were: diagnosis of DM1 or DM2 through genetic testing, based upon the clinical diagnostic criteria set by the International Consortium for Myotonic Dystrophy (30, 31); presence of a complete set of short-axis cine sequences; presence of both native and post-contrast T1 maps. We excluded patients who had a low (≤50%) ejection fraction at echocardiography, abnormal ECG parameters or who presented overt symptoms or signs of cardiac pathology and thus would not represent the ideal population for screening early cardiac damage.

### Protocol approval and informed consent

The study was conducted in accordance with the Declaration of Helsinki, and approved by the Local Ethics Committee Comitato Etico Milano area 2 (protocol code “FibroRetro,” number 1042_2022).

### Neuromuscular and cardiological assessment

Neurological evaluation and chart reviews was performed by neurologists experienced in neuromuscular diseases. Personal and family history was investigated and standard clinical information was obtained. Muscle strength was assessed manually using the standard medical research council (MRC) scale. Single muscular group and global strength were manually quantified according to the medical research council (MRC) scale, grading strength from 0 to 5 in each one, applied to 28 muscular groups, for a score total of 140 (32). Functional impairment in DM1 patients was also evaluated using the muscular impairment rating scale (MIRS) (33).

According to clinical practice, all patients had undergone basal 12-lead electrocardiogram (ECG) with surface electrodes and a 24-h Holter- ECG to detect potential cardiac involvement. The duration of QRS complex, PR, QTc interval, and eventual bouts of atrial fibrillation were also registered. A transthoracic 2D-echocardiogram had been performed in DM1 patients, and data regarding the ejection fraction was collected.

DM1 and DM2 genotyping was performed on genomic DNA extracted from peripheral blood leukocytes (24).

### Image acquisition and assessment

A 1.5 T (Siemens, Sonata) CMR was performed including kinetic sequences, T1 mapping and inversion recovery for the LGE after administration of contrast (0.1 mmol/kg of gadobutrol, Gadavist, Bayer).

Regions of interest (ROI) were drawn in the left ventricle for blood T1 measurement and in all the ventricle wall in a four chamber and two chamber view. We used T1 data obtained before and after contrast injection. ECV was calculated. We also measured the volume and function of both ventricles segmenting the right and left ventricle subendocardium with a dedicated software (MEDIS QMass 7.6, Medical Imaging Systems). For the segmentation of cardiac images, the reader manually traced epicardial contour at both the end-diastolic and end-systolic phases. Then, a blood thresholding technique (Mass-K mode) was applied, and end-diastolic volume index with regards to body surface area (EDVI), end-systolic volume (ESVI), stroke volume (SV), and ejection fraction (EF) were calculated automatically. A 50% threshold was fixed using Mass-K mode. LGE was visually evaluated.

### Statistical analysis

Normal data distributions were reported as mean ± standard deviation, while non-normal data distributions were reported as median and interquartile range (IQR). The minimum and the maximum value of each distribution were also reported. Differences between independent distributions were evaluated using Mann–Whitney U test, while correlations were evaluated using Spearman’s *ρ*. The t-test was used to compare the global ECV obtained in our DM1 patients with that published in literature by Sardanelli et al. in healthy subjects (34). Statistical analysis was performed using GraphPad Prism, and *p*-values lower than 0.05 were considered as significant.

## Results

### Demographic and clinical data

A total of 18 DM1 patients (9 females and 9 males) and 4 DM2 patients (2 males and 2 females) were analyzed. The median age at onset of DM1 in our patients was 30 (IQR 23–35) years, and the median age at the day of the examination was 31 (IQR 24–37) years. Conversely, the median age at onset of DM2 patients was 31 (IQR 21–40) and the median age at review was 47.5 (IQR 34–52). In DM1 patients, age at review, age at onset and MIRS were homogeneous between males and females (Table 2). Ten patients with DM1 had *DMPK* expansion in the E1 range (50–150 repeats), eight patients had *DMPK* expansion in the E2 range (151–1,000 repeats). Two patients could be classified as mild DM1, while the remaining 16 patients were classified as classic DM1. As regards DM2 patients, two patients had marked myotonic phenomenon, one of then associated with myalgias and endocrine disturbances. Two patients, conversely, displayed a proximal myotonic myopathy (PROMM) phenotype, with proximal weakness; one patient also reported myalgias, while the other patients developed bilateral cataracts. Distribution characteristics of our DM1 and DM2 patients are reported in Tables 2, 3. We assessed cardiac function using traditional examination methods, namely transthoracic echocardiography and electrocardiographic evaluation. All our patients demonstrated normal parameters of cardiac function and conduction (Table 3).

**Table 2:** Demographic breakdown according to gender in DM1 patients.

	Females (*n* = 9)	Males (*n* = 9)
Age at review, *median (IQR)*	30 (25–33)	36 (25–56)
Age at onset, *median (IQR)*	30 (25–33)	30 (16.5–43.5)
MIRS scale, *median (range)* (DM1 patients)	2 (1–4)	2 (1–4)

**Table 3:** Demographic and clinical data of our patient cohort.

	DM1 (*n* = 18)	DM2 (*n* = 4)
Females	9 (50%)	2 (50%)
Age at review, *median (IQR)*	31 (24–37)	47.5 (34–52)
Age at onset, *median (IQR)*	30 (23–35)	31 (21–40)
MIRS scale, *median (range)* (DM1 patients)	2 (1–4)	/
MRC total score, *mean ± SD*	136.2 *±* 6.48	137.5 *±* 3.20
Heart rate (bpm), *median (IQR)*	70.50 (65–74.65)	85 (66.25–97.75)
PR interval (ms), *median (IQR)*	168 (155–202.50)	160 (155.50–169)
QRS interval (ms), *median (IQR)*	115 (102.80–151.50)	94 (86.25–99.50)
EF (%), *median (IQR)*	61 (60–67)	70 (66.10–78.33)

bpm, beats per minute; DM, myotonic dystrophy; EF, ejection fraction; IQR, interquartile range; MIRS, myotonia impairment rating scale; MRC, medical research council.

### Imaging assessment

**Figure 2:**
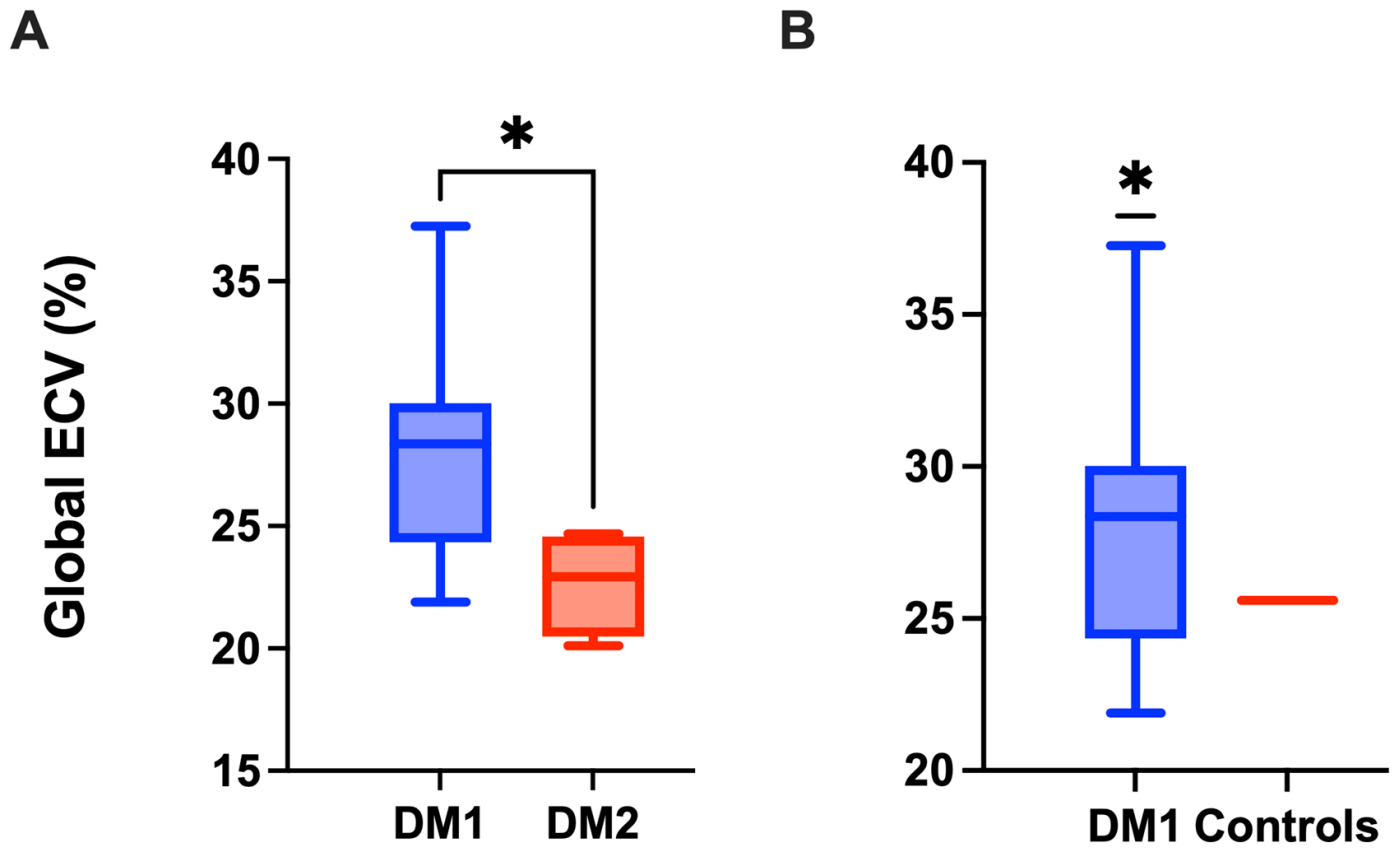
Global extracellular volume (ECV) is increased in DM1 patients compared to DM2 patients **(A)** and healthy subjects **(B)**. **(A)** Global ECV. Data are represented as median ± IQR for DM1 and DM2 patients (Mann–Whitney U-test, *p < 0.05). **(B)** Global ECV. Data are represented as median ± IQR for DM1 patients and as median value for healthy controls (25.60) as from Sardanelli et al. (34) (One sample t-test, *p < 0.05).

**Figure 3:**
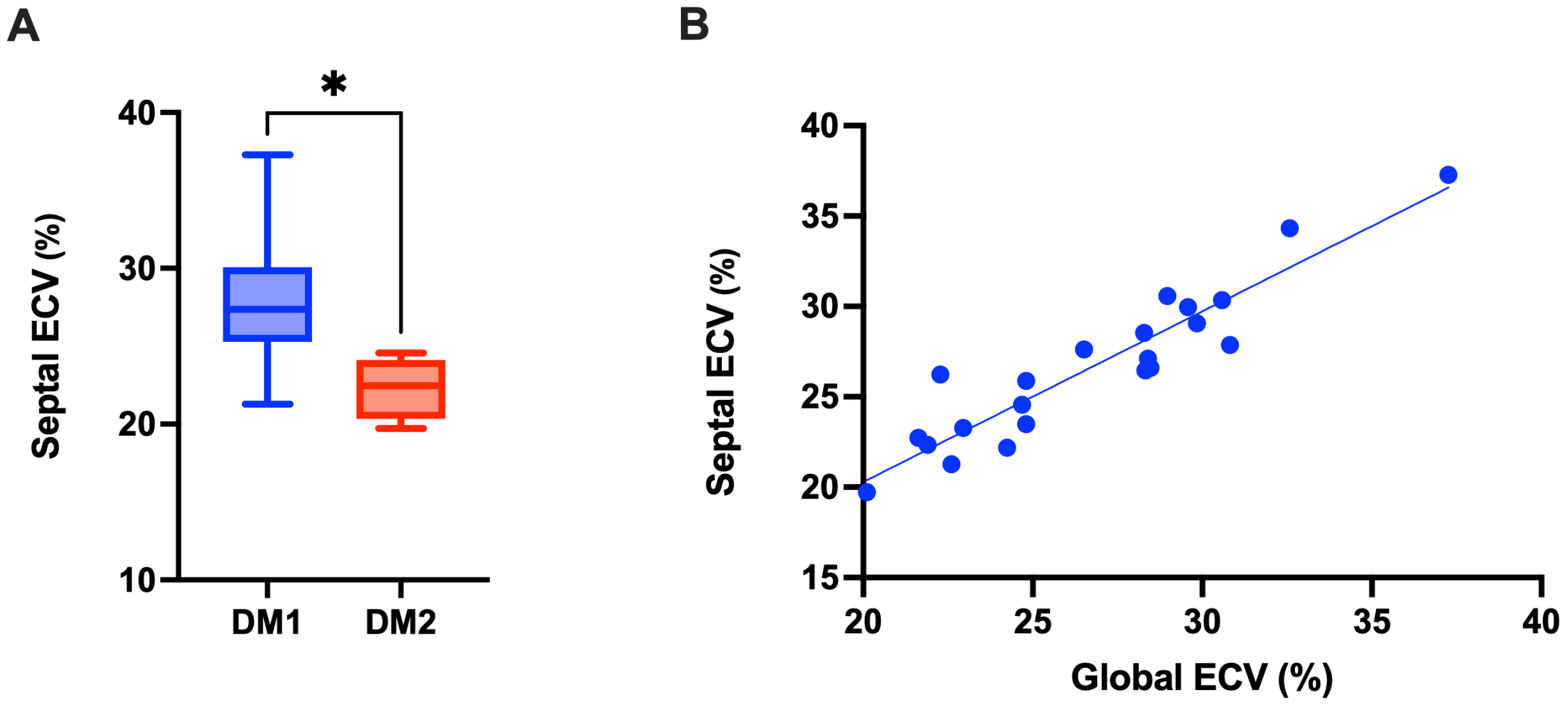
Septal ECV values correlate with global ECV values and are significantly increased in DM1 compared to DM2 patients. **(A)** Septal ECV. Data are represented as median ± IQR for DM1 and DM2 patients (Mann–Whitney U-test, *p < 0.05). **(B)** Correlation between Septal ECV and global ECV in pooled DM1 and DM2 patients (Spearman r test; *ρ* = 0.9099, *p* < 0.0001).

We performed CMR examination in our patient cohort as described in the Methods’ section. In all the patients no LGE was reported. Some traditional CMR parameters proved slightly reduced compared to reference values in both DM1 and DM2 populations, with preservation of left ventricle ejection fraction (EF) in both groups (Table 4). Concerning ECV, median global ECV was 28.36% (IQR 24.81–29.77) in DM1 patients with values that ranged from 22.60 to 37.26%, while median septal ECV was 27.37% (IQR 25.97–29.74) with values spanning from 21.28 to 37.29%. In DM2 patients, median global ECV was 22.93 (IQR 21.25–24.35) with values that ranged from 20.11 to 24.68%, while median septal ECV was 22.46% (21.57–23.19), with values spanning from 19.73 to 24.37%. The global ECV obtained in our DM1 patients was significantly higher compared to DM2 patients (*p* = 0.0141; Figure 2A) and to values reported in literature for healthy subjects (25.60%, IQR 19.90–31.90%) as from (34) (*p* = 0.0374; Figure 2B). A very strong positive correlation was observed between global ECV and septal ECV (*ρ* = 0.9099, *p* < 0.0001) (Figure 3), suggesting that DM1 patients may be at increased risk of early septal fibrosis.

**Table 4:** Baseline cardiac magnetic resonance findings in male and female patients.

	Reference values for women, *mean ± SD, lower-upper limit*	DM1 patients, *mean ± SD* (*n* = 9)	DM2 patients, *mean ± SD* (*n* = 2)
Women			
LV ESVI (mL/m^2^)	24 ± 5, 14–34	18.05 ± 4.15	10.07 ± 1.71
LV EDVI (mL/m^2^)	76 ± 10, 56–96	55.66 ± 7.83	43.30 ± 1.30
LV SV (mL)	87 ± 15, 57–117	62.89 ± 12.71	60.88 ± 5.69
LV EF (%)	67 ± 5, 57–77	68.23 ± 8.42	76.68 ± 4.67
RV ESVI (mL/m^2^)	32 ± 10, 12–52	14.72 ± 4.29	12.30 ± 3
RV EDVI (mL/m^2^)	80 ± 16, 48–112	47.41 ± 7.11	41.22 ± 0.2
RV SV (mL)	84 ± 18, 48–120	54.08 ± 12.97	52.87 ± 3.84
RV EF (%)	61 ± 5, 51–71	68.41 ± 10.04	70.14 ± 7.48

	Reference values for men, *mean ± SD, lower-upper limit*	DM1 patients, *mean ± SD* (*n* = 9)	DM2 patients, *mean ± SD* (*n* = 2)
Men			
LV ESVI (mL/m^2^)	26 ± 6, 14–38	20.34 ± 5.67	13.61 ± 1.7
LV EDVI (mL/m^2^)	81 ± 12, 57–105	56.80 ± 9.73	43.52 ± 1.26
LV SV (mL)	108 ± 18, 72–144	69.43 ± 13.96	53.99 ± 3.11
LV EF (%)	67 ± 5, 57–77	64.47 ± 7.48	68.78 ± 2.99
RV ESVI (mL/m^2^)	39 ± 10, 19–59	18.46 ± 4.42	21.74 ± 4.83
RV EDVI (mL/m^2^)	184 ± 33, 118–250	53.86 ± 6	49.23 ± 3.55
RV SV (mL)	106 ± 19, 68–144	67.72 ± 11.24	49.66 ± 4.44
RV EF (%)	62 ± 5, 52–72	65.80 ± 6.24	56.09 ± 6.65

Reference values are derived from Kawel-Boehm et al. (48).

ECV, extracellular volume; LV, left ventricle; RV, right ventricle; EDVI, end-diastolic volume index; ESVI, end-systolic volume index; SV, stroke volume; EF, ejection fraction.

**Figure 4:**
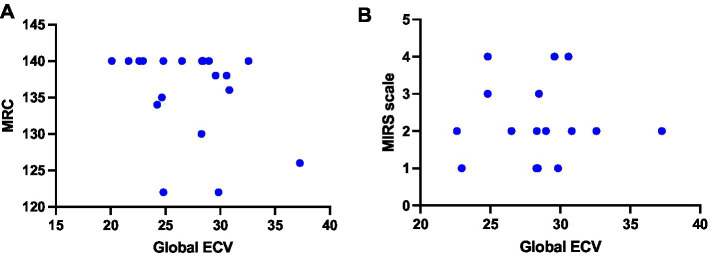
Relationship between ECV values and neuromuscular status of DM1 patients. **(A)** Correlation between global ECV and total MRC scores (Spearman r test; *ρ* = −0.2938, *p =* 0.2087) **(B)** Correlation between global ECV and MIRS scale values (Spearman r test; *ρ* = −0.2938, *p =* 0.2087). ECV, extracellular volume; MIRS, myotonia impairment rating scale; MRC, medical research council.

**Figure 5:**
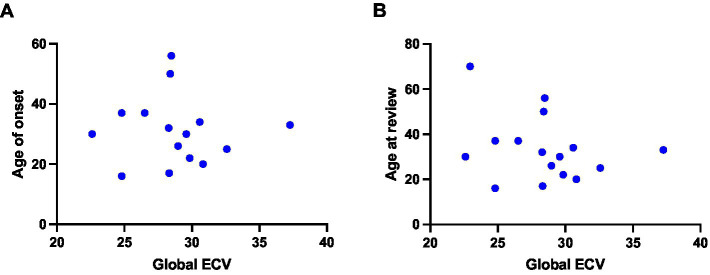
Relationship between ECV values and clinical parameters in DM1 patients. Figure shows correlations between: **(A)** Global ECV and age at onset (Spearman r test; *ρ* = −0.02718, *p =* 0.9234) **(B)** Global ECV and age at review (Spearman *r* test; *ρ* = −0.2395, *p =* 0.3679).

We evaluated whether there was a correlation between ECV values and the degree of neuromuscular impairment in our DM1 cohort, and we observed that ECV values did not correlate with total MRC score nor with MIRS scale values (Figure 4). Furthermore, we observed no correlation with age of symptom onset and age at review (Figure 5). We also evaluated the relationship of ECV values with other parameters of cardiac function. We found that ECV values did not correlate with other CMR-derived measures of cardiac morphology and function, nor with ECG-derived measures of cardiac conduction (PR interval length and QRS interval length) (Supplementary Figure 1).

**Figure 6:**
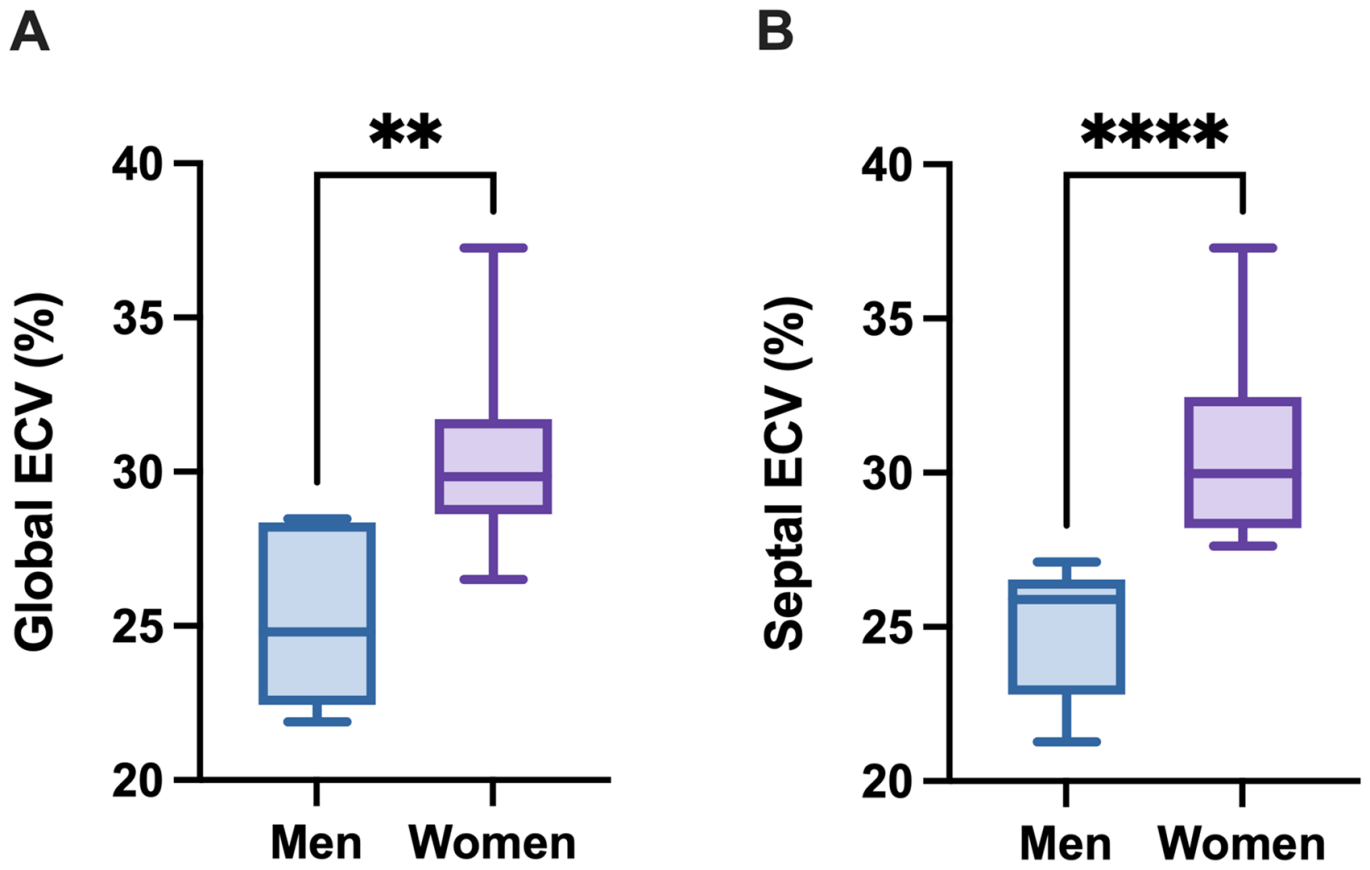
Global and septal extracellular volumes are increased in female DM1 patients compared to male DM1 patients. **(A)** Global ECV. Data are represented as median ± IQR for male and female patients (Mann–Whitney U-test, **p < 0.01). **(B)** Septal ECV. Data are represented as median ± IQR for male and female patients (Mann–Whitney U-test, ****p < 0.0001).

Given the gender-related differences in myotonic dystrophy phenotype reported in the literature (35), we decided to assess differences between men and women in our cohort. We observed that women showed significantly higher global (Figure 6A; *p =* 0.0012) and septal (Figure 6B; *p* < 0.0001) ECV values compared to men.

## Discussion

Our study aimed to evaluate whether ECV may be altered in patients affected by Myotonic Dystrophy type 1 and type 2, in order to investigate its potential as biomarker of subclinical cardiac involvement. ECV has been studied as a biomarker of cardiac fibrosis, with increased ability to capture myocardial damage not apparent at traditional cardiac assessment.

In our cohort, we observed significantly increased ECV values in DM1 patients compared to DM2 patients and reference values in healthy controls. With all the limitations connected with the nature of the study and the width of the sample, this finding suggest the presence of a certain degree of myocardial fibrosis in the left ventricle of DM1 patients. Previous studies yielded somewhat conflicting results (Table 1), but overall showed an increase in ECV values compared to controls and/or DM2 patients when different portions of the left ventricle were sampled (24, 26, 27). A notable exception is the paper by Chmielewski and colleagues which observed an increase in LGE, mainly found in the mid-wall layer of septal and inferolateral segments, but not in global ECV (18). Our study confirms previous reports of increased ECV values and subclinical fibrotic processes in DM1 patients.

In addition to global ECV, we decided to measure septal ECV also since its higher thickness makes ECV calculation more accurate in the septal area. The punctual correlation between global and septal ECV adds robustness to our data. It may also indicate that fibrotic fenomena are prevalent in the interventricular septum. This observation may have important clinical consequences as septal fibrosis has been associated with increased risk of atrioventricular arrhythmias and blocks and sudden cardiac death (26, 36). Furthermore, previous autoptical studies conducted on DM1 patients revealed that fibrosis was more marked in the septum compared to other left ventricular zones (11). Indeed, Leali and colleagues found a progressive increase over time in septal wall thickness measured with CMR in DM1 patients, and a positive correlation between septal wall thickness and implantation of pacemaker (PM)/implantable cardiac defibrillator (ICD) devices at follow-up (26). These findings hint at a potential progressive process going on within the interventricular septum in this category of patients, and reinforce the association between septal fibrosis and risk for fatal arrhythmias. Indeed, septal fibrosis seem to create an environment that is prone to the development of conduction diseases, even though the exact interplay and relationship between this event still needs to be elucidated (5, 18, 37, 38). In a previous study, Petri and colleagues report the absence of an association between myocardial fibrosis, measured with increased LV mass, increased left atrial volume and reduced LVEF at CMR and ECG abnormalities. On the opposite, Hermans and colleagues found a strong association between the presence of LGE, often located in the interventricular septum, and ECG abnormalities. Similarly, Chmielewski and colleagues observed a relationship between presence of LGE and detection of rhythm and/or conduction abnormalities at Holter-ECG recordings (18). In the present study, we did not retrieve a statistically significant correlation between global and septal ECV and ECG-derived measures of cardiac conduction. This could be due to the small dimension of our cohort. Another potential explanation lies in the good cardiovascular status of the patients that we analyzed, since all of them were asymptomatic from a cardiac point of view and had normal ECG, while statistically significant associations in the abovementioned papers were observed in case of overt conduction disturbances (rhythm other than sinus, abnormal values of PR or QRS intervals, conduction blocks). In addition, ECV values of our patients do not correlate with traditional CMR parameters of cardiac function, thus suggesting that subclinical fibrosis may precede cardiac function decline (Supplementary Figure 1).

Abnormal ECV values were independent of the presence of neuromuscular symptoms, as already observed previously (18). In a previous study of CMR in DM patients, Alì and colleagues described a trend toward a positive correlation between global ECV and MIRS scores, although it did not reach statistical significance (24). This finding suggest that subclinical cardiac impairment does not match neuromuscular performance status, and further reinforces the need for early cardiac screening also for asymptomatic and mildly affected subjects.

Notably, we observed significantly increased values of both global and septal ECV in DM1 women compared to DM1 men. Previously, ECV values were found to be higher in women compared to men in several literature cohorts (39–43). These studies underline the presence of a relationship between gender and cardiovascular architecture and health. This could be due to differences in body composition (44). Additionally, sex hormones have been found to influence myocardial structure, function and histology (45). Interestingly, gender-related differences in the proteome of patients with heart failure were observed, with differences in the baseline levels of circulating proteins related to extracellular matrix organization, higher in women, and regulation of cell death, higher in men (46). The same study also found differences in serial measurements of the circulating proteins endothelin-1 and somatostatin, and an association of these values with adverse cardiovascular outcome. These observations suggest that there may be both physiological differences in myocardial architecture and function in healthy status, with extracellular tissue being more represented in women, and different responses to risk factors and cardiac pathological processes. As regards myotonic dystrophies, recent studies have pointed out the influence of gender on phenotype severity and prevalence of multisystemic manifestations (35, 47). Dogan and colleagues found a higher prevalence of cardiac manifestations in men compared to women, although the retrospective nature of their study and limited data does not allow to assess the impact of risk factors on this finding (35). Our results, taken together with literature data, suggest that women may be more prone than their male counterparts to develop myocardial fibrosis, thereby warranting a strict monitoring of cardiac function. Additional clinical and instrumental data are necessary to better assess the impact of gender on cardiac function.

Our study also presents significant limits. The first is surely the limited size of the screened sample; significantly higher numbers are necessary in order to draw a more precise picture of the cardiovascular profile of DM1 and 2 patients with CMR. Another main limitation is the absence of a control group. However, several studies and meta-analyses in literature have already assessed ECV values in healthy subjects. We used values derived from a published meta-analysis on ECV values in healthy subjects, measured with the same machine used in our study (34). The small sample size and lack of control group are mainly due to the limited availability of CMR and the high costs of the exam. However, CMR is now gaining increased popularity and is increasingly used in clinical practice, therefore raising hopes for the possibility to include more patients, alongside with healthy controls, in future studies.

In conclusion, our study underscores the added value of CMR in detecting subclinical myocardial changes in DM1 patients. As for now, recommendations from the American Heart Association include baseline and periodic (every 1–5 years) monitoring of asymptomatic DM patients with cardiac imaging, either echocardiography with strain imaging or CMR. Results from this and previous studies suggest that, when available, CMR should be considered as a screening measure in this patient population due to its possibility to detect subclinical myocardial involvement, that can be overlooked by ECG and echocardiography alone, and with important consequences on therapeutic management and timing for follow-up. More specifically, DM patients with structural or functional heart disease may be eligible for treatment with beta-adrenergic blockers, angiotensin-converting enzyme inhibitors, or angiotensin-receptor blockers (4). In addition, patients at high risk of arrhythmic events at non-invasive testing may be eligible to invasive electrophysiology testing and/or to pacemaker or ICD positioning (4). CMR detection of subclinical cardiac disease may thus have an important impact on the definition of prognosis and in the consequent eligibility for life-saving procedures. Further studies are needed to better define the role of CMR measurements, such as ECV, in the clinical management of patients. This approach is in line with the recent findings and recommendations, suggesting a paradigm shift toward more sensitive and comprehensive cardiac evaluation methodologies in patients with Myotonic Dystrophy.

## Data Availability

The raw data supporting the conclusions of this article will be made available by the authors, without undue reservation.
