# Supplementary material for: Cardiac risk and myocardial fibrosis assessment with cardiac magnetic resonance in patients with myotonic dystrophy
Source: Front Neurol. 2024 Nov 21;15:1493570. doi: 10.3389/fneur.2024.1493570 (PMC11617365; doi:10.3389/fneur.2024.1493570)
Supplement: Supplementary file 1 [file Table_1.DOCX]

**Supplementary Material.**

**Supplementary Figure 1.** Relationship between ECV values and measures of cardiac morphology and function and of cardiac conduction in DM1 patients. Figure shows correlations between: A) Global ECV and LV ESVI (Spearman r test; ρ = 0.3498, *p=* 0.1306) B) Global ECV and LV SV (Spearman r test; ρ = -0.1361, *p=* 0.5671) C) Global ECV and LV EDVI (Spearman r test; ρ = 0.2221, *p=* 0.3467) D) Global ECV and LV EF (Spearman r test; ρ = -0.2633, *p=* 0.2621) E) Global ECV and RV ESVI (Spearman r test; ρ = 0.1053, *p=* 0.6586) F) Global ECV and RV SV (Spearman r test; ρ = -0.3422, *p=* 0.1397) G) Global ECV and RV EDVI (Spearman r test; ρ = -0.1941, *p=* 0.4123) H) Global ECV and RV EF (Spearman r test; ρ = 0.000, *p=* >0.9999)

Abbreviations: *LV, left ventricle; RV, right ventricle; EDVI, end-diastolic volume index; ESVI, end-systolic volume index; SV, stroke volume; EF, ejection fraction.*
